# Bullying and Suicide Attempts Among US High School Students

**DOI:** 10.1001/jamanetworkopen.2025.52089

**Published:** 2026-01-02

**Authors:** Khushboo R. Agarwal, Sasha A. Fleary, Philip Kreniske, Chloe A. Teasdale

**Affiliations:** 1 Department of Epidemiology and Biostatistics, Graduate School of Public Health and Health Policy, City University of New York (CUNY), New York, New York; 2 Department of Community Health and Social Sciences, Graduate School of Public Health and Health Policy, CUNY, New York, New York; 3 Institute for Implementation Science in Population Health, CUNY, New York, New York

## Abstract

This cross-sectional study evaluates the association of experiencing bullying on school grounds with likelihood of suicide attempt among participants in the 2023 Youth Risk Behavior Surveillance System.

## Introduction

Bullying is associated with increased risk of suicidality in adolescents.^1^ Female adolescents report higher rates of bullying and suicidal behaviors.^1^ To identify adolescents at high risk for bullying-related suicide attempt, we examined school-based bullying and suicide attempts and assessed modification by sex.

## Methods

For this cross-sectional study, we analyzed data from the 2023 Youth Risk Behavior Surveillance System (YRBSS), a cross-sectional, school-based survey conducted annually in all 50 US states.^2^ The present analysis was deemed nonhuman participant research by the CUNY Institutional Review Board because deidentified publicly available data were used. We followed the STROBE reporting guideline.

The outcome was self-reported suicide attempt in the past 12 months. The exposure was school-based bullying, which was measured using the question, “During the past 12 months, have you ever been bullied on school property?” Confounders included age, sex, race and ethnicity, sexual identity (lesbian, gay, bisexual, queer, and questioning [LGBQ+]; heterosexual or straight), and gender identity (transgender; not transgender). Multivariable models, adjusted for confounders, were used to estimate the association between bullying and suicide attempt. Missing responses for variables were included but not reported from models.

We used an interaction term to assess multiplicative effect measure modification (EMM) by sex of the association between bullying and suicide. On the additive scale, EMM was assessed using multivariable logistic regression models (including confounders) to estimate relative excess risk due to interaction (RERI).^3^ Participants who reported no bullying (unexposed) and were male (unmodified) were the common reference group for estimating adjusted odds of suicide attempt for bullied males (exposure only), nonbullied females (modifier only), and bullied females (exposed and modified).

Two-sided *P* < .05 indicated statistical significance. Data analysis was conducted from April to July 2025 using SAS Studio 3.81 (SAS Institute).

## Results

Among the 20 103 participating high school students (10 061 males [51.9%]; 10 312 [51.3%] aged 15-16 years), 19.2% reported being bullied at school and 9.5% reported past-year suicide attempt. Bullying was reported by higher proportions of younger students, females, LGBQ+ youth, and transgender individuals (Table 1).

**Table 1.** YRBSS Participant Characteristics Overall and Stratified by Bullying Status

Characteristic	All		Bullied		Nonbullied		*P* value^a^
	Unweighted No.	Weighted % (95% CI)	Unweighted No.	Weighted % (95% CI)	Unweighted No.	Weighted % (95% CI)	
No.	20 103	NA	4046	19.2 (17.2-21.3)	15 856	80.8 (78.7-82.8)	NA
Age group, y							
12-14	2646	12.6 (11.0-14.2)	648	23.5 (20.1-26.8)	1963	76.5 (73.2-79.9)	<.001
15-16	10 734	51.3 (49.5-53.1)	2302	20.9 (18.6-23.2)	8324	79.1 (76.8-81.4)	
≥17	6625	36.1 (34.1-38.2)	1082	15.5 (13.1-17.8)	5493	84.5 (82.2-86.9)	
Missing responses	98	NA	NA	NA	NA	NA	NA
Sex at birth							
Male	10 061	51.9 (49.7-54.0)	1663	16.6 (14.7-18.6)	8311	83.4 (81.4-85.3)	<.001
Female	9884	48.1 (46.0-50.3)	2338	21.9 (19.3-24.5)	7453	78.1 (75.5-80.7)	
Missing responses	158	NA	NA	NA	NA	NA	NA
Race and ethnicity^b^							
Hispanic or Latino^c^	3994	27.4 (22.5-32.3)	136	15.8 (13.1-18.6)	1062	84.2 (81.4-86.9)	<.001
Non-Hispanic American Indian or Alaska Native and non-Hispanic Asian or Pacific Islander^d^	2434	5.0 (3.1-6.9)	377	11.5 (7.9-14.9)	2037	88.6 (85.0-92.0)	
Non-Hispanic Black or African American	1791	13.3 (8.5-18.1)	252	14.2 (11.9-16.5)	1521	85.8 (83.5-88.1)	
Non-Hispanic White	9700	48.2 (41.4-54.9)	2255	23.2 (20.9-25.5)	7372	76.8 (74.5-79.1)	
Non-Hispanic multiracial	1814	6.1 (4.2-8.1)	449	21.5 (15.5-27.4)	1356	78.5 (72.6-84.5)	
Missing responses	370	NA	NA	NA	NA	NA	NA
Sexual identity							
LGBQ+	4346	24.2 (22.0-26.4)	1306	28.7 (24.9-32.6)	3004	71.3 (67.4-75.1)	<.001
Heterosexual or straight	13 289	68.8 (71.2-75.5)	2212	15.7 (14.3-17.2)	11 012	84.3 (82.9-85.7)	
Did not understand this question	465	2.5 (2.0-3.0)	91	14.2 (8.6-19.8)	371	85.8 (80.3-91.4)	
Missing responses	2003	NA	NA	NA	NA	NA	NA
Gender identity							
Transgender^e^	612	3.3 (2.7-3.9)	277	42.6 (34.9-50.3)	328	57.4 (49.7-65.1)	<.001
Not transgender	16 986	93.2 (92.4-94.1)	3129	17.3 (15.8-18.8)	13 736	82.7 (81.2-84.2)	
Not sure if I am transgender	428	2.2 (1.7-2.6)	156	38.8 (30.3-47.3)	263	61.2 (52.8-69.7)	
Did not understand this question	275	1.3 (1.0-1.6)	62	24.8 (14.6-35.0)	209	75.2 (65.0-85.4)	
Missing responses	1802	NA	NA	NA	NA	NA	NA
Attempted suicide							
Yes	1995	9.5 (8.3-10.6)	836	43.9 (38.6-49.1)	1123	56.1 (50.9-61.4)	<.001
No	17 341	90.5 (89.4-91.7)	3005	16.1 (14.7-17.5)	14 217	83.9 (82.5-85.3)	
Missing responses	767	NA	NA	NA	NA	NA	NA

Abbreviations: LGBQ+, lesbian, gay, bisexual, queer, and questioning; NA, not applicable; YRBSS, Youth Risk Behavior Surveillance System.

^a^ Rao Scott χ^2^ test accounted for survey weights.

^b^ Self-reported by participants. Race and ethnicity were collected to ensure that the survey was representative of the population of US high school students and to create accurate survey weights.

^c^ Includes Hispanic, Latino, and multiple Hispanic ethnicities.

^d^ Two categories were combined due to small sample sizes for each group.

^e^ Assessed using the questionnaire item, “Some people describe themselves as transgender when their sex at birth does not match the way they think or feel about their gender.”

Participants who reported school bullying had higher adjusted odds of suicide attempt compared with those who were not bullied (adjusted odds ratio [AOR], 3.58; 95% CI, 2.95-4.34) (Table 2). We found evidence of EMM on the additive scale: compared with nonbullied males, bullied females had the highest adjusted odds of suicide attempt (AOR, 5.65; 95% CI, 4.42-7.22) compared with bullied males (AOR, 3.25; 95% CI, 2.29-4.62) and nonbullied females (AOR, 1.50; 95% CI, 1.18-1.89). The RERI was 1.90 (95% CI, 0.55-3.24) (Table 2).

**Table 2.** Multivariable Model of Overall Association of Bullying on School Grounds With Attempted Suicide, and Assessment of Effect Modification by Sex (N = 16 948)

Status	AOR (95% CI)			RERI (95% CI)^a^
	Overall	Male	Female	
Bullied	3.58 (2.95-4.34)	3.25 (2.29-4.62)	5.65 (4.42-7.22)	1.90 (0.55-3.24)
Nonbullied^b^	1 (Reference]	1 (Reference]	1.50 (1.18-1.89)	

Abbreviations: AOR, adjusted odds ratio; RERI, relative excess risk due to interaction.

^a^ RERI estimated with 95% CI to measure effect measure modification on the additive scale.

^b^ For overall estimates, nonbullied status was the reference group. For analysis of additive effect measure modification, nonbullied males were the common reference group.

## Discussion

In this representative sample of US high school students, we found further evidence that bullying was associated with increased likelihood of suicide attempt and identified that female students may be disproportionately affected by bullying. While some evidence-based interventions have been identified to address adolescent suicide and bullying individually, few interventions target both or are developed for the school setting.^4^ This gap is critical given findings from a meta-analysis showing that most suicide and self-injury interventions did not significantly reduce suicidal thoughts and behaviors.^5^ There is an urgent need for integrated approaches addressing both bullying and mental health of affected youth.

A study strength is the assessment of EMM by sex on the additive scale, which showed 5.65-times higher odds of suicide attempt in female participants who reported bullying. A study limitation is that the survey did not measure severity of bullying and did not include adolescents who died by suicide. Nationally, adolescent male suicide death rates exceed those for females; in 2022, the rate was 17.3 per 100 000 for males 15 to 19 years and 6.4 per 100 000 for their female counterparts.^6^ Some of the differential outcomes in females we observed may be due to the inability to account for male suicide deaths. However, given the higher rate of suicide attempts in female participants, our findings reflect new and important evidence that can help inform more effective intervention efforts.

## Notes

Supplement.
Data Sharing Statement
