# Supplementary material for: Bullying and Suicide Attempts Among US High School Students
Source: JAMA Netw Open. 2026 Jan 2;9(1):e2552089. doi: 10.1001/jamanetworkopen.2025.52089 (PMC12761328; doi:10.1001/jamanetworkopen.2025.52089)
Supplement: Supplement. — Data Sharing Statement [file jamanetwopen-e2552089-s001.pdf]

Data Sharing Statement

Agarwal. Bullying and Suicide Attempts Among US High School Students. *JAMA Netw Open*. Published January 02, 2026. doi:10.1001/jamanetworkopen.2025.52089

Data
Data available: Yes
Data types: Deidentified participant data
How to access data: https://www.cdc.gov/yrbs/media/pdf/2023/2023_National_YRBS_Data_Users_Guide508.pdf
When available: With publication

Supporting Documents
Document types: None

Additional Information
Who can access the data: Publicly available dataset
Types of analyses: any
Mechanisms of data availability: Publicly available dataset
Any additional restrictions: None
